# Abnormal Visual Function Outside the Area of Atrophy Defined by Short-Wavelength Fundus Autofluorescence in Stargardt Disease

**DOI:** 10.1167/iovs.61.4.36

**Published:** 2020-04-25

**Authors:** Janet S. Sunness, Abraham Ifrah, Robert Wolf, Carol A. Applegate, Janet R. Sparrow

**Affiliations:** 1 Richard E. Hoover Low Vision Rehabilitation Services and Department of Ophthalmology, Greater Baltimore Medical Center, Baltimore, Maryland, United States; 2 Department of Ophthalmology and Visual Sciences, University of Maryland School of Medicine, Baltimore, Maryland, United States; 3 Lewis Katz School of Medicine at Temple University, Philadelphia, Pennsylvania, United States; 4 Boston University School of Medicine, Boston, Massachusetts, United States; 5 Department of Population Medicine, Harvard Medical School/Harvard Pilgrim Health Care Institute, Boston, Massachusetts, United States; 6 Department of Ophthalmology, Harkness Eye Institute, Columbia University Medical Center, New York, New York, United States

**Keywords:** Stargardt, microperimetry, fundus autofluorescence

## Abstract

**Purpose:**

To examine the extent of visual function abnormality outside the dark lesion on short-wavelength fundus autofluorescence (SW-AF), and its correlation with background SW-AF features and optical coherence tomography (OCT) in recessive Stargardt disease (STGD1)

**Methods:**

Forty-nine eyes of 25 participants in the ProgStar (the Natural History of the Progression of Atrophy Secondary to Stargardt Disease) study at our center were included. Patients underwent microperimetry (both threshold and dense scotoma mapping), OCT, SW-AF, and visual acuity testing. The Fisher's exact test, the χ^2^ test, and unpaired *t*-tests were used to analyze the data.

**Results:**

Of 40 eyes without central fixation, 33 (82%) placed fixation remote (most ≥5°) from the dense scotoma edge, despite good intervening retinal sensitivity. OCT findings accounted for the remote fixation in 75%. Eighteen (37%) of all 49 eyes had dense scotoma extending past the dark lesion border. OCT was not adequate to define the edge of the scotoma. Of the 49 eyes, 28 (57%) had the mottled background pattern, 10 (20%) had the uniform pattern, and 11 (22%) had the other pattern, with >75% of eyes in each pattern having remote fixation. The dense scotoma exceeded the dark lesion primarily in the mottled pattern. The two eyes of each patient were concordant in all features.

**Conclusions:**

Functional abnormalities in STGD1 extend past the SW-AF dark lesion. The disruption of the ellipsoid zone shows that photoreceptor abnormality extends peripheral to the dark lesion, and it explains in part the remote fixation pattern and the dense scotoma exceeding the dark lesion. This has implications for clinical trials for STGD1.

Autosomal recessive Stargardt disease (STGD1) is the most common cause of macular degeneration in young people. The *ABCA4* gene was discovered to be the gene responsible for STGD1 in 1997.^1^ The *ABCA4* gene product functions as a transporter protein,^2^ without which the end result is the accumulation of excessive lipofuscin in the retinal pigment epithelial (RPE) cells.^3^ This accumulation may be toxic to the RPE in the long term, and at some critical time, often the same for each eye, the RPE and overlying photoreceptors begin degenerating. The knowledge gained over the last 2 decades has led to gene therapy trials (NCT01345006 and others),^4^ clinical trials associated with deuterated vitamin A that may reduce bisretinoid accumulation (NCT02402660),^5^ and clinical trials of visual cycle modulators (NCT03772665),^6^ that reduce the production of bleached retinoids that would ultimately form bisretinoid fluorophores of lipofuscin, including A2E.

However, it is known that there is extensive lipofuscin accumulation in the RPE as early as 13 months of age in patients who go on to develop clinical STGD1 later in life.^7^ In fact, the presence of a “dark choroid” on fluorescein angiography, associated with blockage of underlying fluorescence by lipofuscin, is a hallmark of STGD1. It is unknown what triggers the onset of RPE degeneration in the longstanding presence of extensive lipofuscin accumulation. The onset of degeneration is often similar in both eyes of an individual, and is often similar in siblings with the same genotype, so it is possible that it is a factor other than the precise amount of accumulation that spurs the onset of disease.

It is possible then that decreasing the further accumulation of lipofuscin, whether by gene therapy, visual cycle modulators, alternative vitamin A, or other means, will not have an impact on RPE atrophy and on the progression of the disease.

There is one way in which treatments based on limiting further bisretinoid accumulation would have a definite impact on the disease. If the bisretinoids in the outer segment exert a direct toxic effect on the photoreceptors themselves, then a treatment that decreases the accumulation of bisretinoids in real-time could have a beneficial effect on photoreceptor viability and function. One can glean some information from the *Abca4* knockout mouse model because the mice exhibit both accelerated bisretinoid accumulation and light-associated photoreceptor damage.^8^^,^^9^ We,^10^^–^^13^ and others,^14^ have shown that the dense scotoma often far exceeds the homogeneous dark area on short-wavelength fundus autofluorescence (SW-AF), so that measuring the homogeneous dark area is not capturing the full extent of the scotoma present. The data reported herein provide strong evidence that severe photoreceptor dysfunction precedes atrophy defined by a loss of SW-AF. It may be that dark areas on near-infrared autofluorescence (NIR-AF), which are typically larger than the dark area on SW-AF, may be a better measure of RPE atrophy and photoreceptor dysfunction, but it is not known how well it corresponds to dense scotomas on microperimetry. The dark lesion on fundus autofluorescence represents the late stage, in which there is loss of both the RPE and photoreceptors; in areas that are not dark, the various analyses of optical coherence tomography (OCT) and visual function are likely to be more informative.

We also have shown that the fixation pattern in many patients with STGD1 is different from essentially all other atrophic macular disorders. In geographic atrophy associated with age-related macular degeneration, fixation is placed, as expected, at a site of functional retinal cells at the edge of the dense scotoma.^11^ In contrast, in STGD1, fixation is often remote from the edge of the scotoma, leading to the patient using a retinal locus much further from the fovea than would seem to be necessary.^11^^,^^13^^,^^15^ This study shows that the intervening seeing retina often has good retinal sensitivity, so a more subtle photoreceptor abnormality may be operant in causing a more peripheral site to be chosen for fixation. This distancing of fixation from the dense scotoma edge effectively enlarges the scotoma by having the patient use a more eccentric fixation position with a consequent lower visual acuity than might be achieved by fixation at a closer site.^16^

The ProgStar (the Natural History of the Progression of Atrophy Secondary to Stargardt Disease) study (NCT 01977846) was a 2-year natural history study of STGD1 disease that included 238 patients from 9 clinical centers. Patients underwent microperimetry, visual acuity testing, SW-AF and OCT imaging every 6 months.^17^ In our center, we supplemented the protocol microperimetric testing^18^ with dense scotoma mapping^10^ to define the full extent of the dense scotoma when it exceeded the grid used for testing. This study afforded us the opportunity to assess retinal function outside the area of central atrophy and loss of autofluorescence, and to determine to what extent photoreceptor function is compromised in retinal areas unexpected to have functional loss based on the SW-AF. We correlate this functional loss with SW-AF patterns and OCT findings. These data may help for future design of clinical trials for STGD1.

## Methods

### Subjects

The subjects of this study were the 26 patients with STGD1 enrolled in the prospective ProgStar study at the Greater Baltimore Medical Center (GBMC) clinical center. Each patient underwent genetic testing and had either two or more *ABCA4* mutations detected, or had one *ABCA4* mutation detected with a classical Stargardt phenotype. Of the 52 eyes with STGD1, two eyes of one patient and one eye of another had unreliable microperimetry (high false-positive rate, inconsistent results); 49 eyes of 25 patients are therefore included in this analysis. The study was approved by the GBMC institutional review board, and informed consent was obtained from all participants. The research adhered to the tenets of the Declaration of Helsinki.

### Procedures

The subjects underwent a baseline evaluation, and then an evaluation every 6 months for 24 months. The study design has been described in detail elsewhere.^16^ Briefly, at each visit, a protocol refraction was performed and best-corrected ETDRS (Early Treatment Diabetic Retinopathy Study) visual acuity was obtained. The eyes were dilated, and the patient underwent MP-1 (Nidek Technologies, Padova, Italy) microperimetry using a Humphrey visual field 10-2 pattern and a 4-2 staircase to determine the threshold at each point tested. The pattern was centered on the fovea regardless of the fixation site, initially by estimating its location by referring to separate OCT images, and later by inputting an OCT image with the fovea marked. In either case, the fixation cross was positioned so as to center the fovea within the MP-1 field. The fixation site and stability were determined based on the fixation during the 10-2 testing. A 2° red fixation cross was used for all patients. The patient underwent Heidelberg Spectralis (Heidelberg Engineering, Heidelberg, Germany) 488 nm SW-AF and OCT imaging. The 488 nm SW-AF image was imported into the MP-1 and registered with the MP-1 image, allowing the stimulus points to be overlaid on the SW-AF image. As part of the OCT testing, 49 high-definition horizontal-line scans were obtained, spanning 6 × 6 mm on the retina. The OCT imaging was analyzed to find the horizontal scan closest to the lesion that showed a fully intact ellipsoid zone (EZ) (called first intact line [FIL]). This analysis was done without access to the microperimetric findings. This FIL analysis was done independently by two authors (JSS and AI), with adjudication of any differences. The OCT image was then registered with the SW-AF microperimetry image in Adobe Photoshop (Adobe, San Jose, CA, USA), and the FIL scan line was transferred onto the image. The relation of this position to the edge of the excess dense scotoma and to the remote fixation were analyzed.

At the GBMC center, dense scotoma mapping was also performed, as described previously.^10^ A semiautomated mode of testing was used first, in which the operator defines a polygonal area to be tested and can specify the number or density of points to be tested. The brightest stimulus (0 dB) was used to test the points in this grid automatically. Additional points are added manually to test the full extent of the scotoma, and to intensively map the border between seeing and nonseeing retina. This testing is particularly necessary when the scotoma extends past the edge of the 10-2 grid; the border of the dense scotoma would remain undefined without the additional testing. Many additional spots were placed to define the border, but because this was only done with one stimulus intensity, the testing was more rapid than for the threshold testing, and was less exhausting for the patient.

After the microperimetry testing, the SW-AF image was imported, and the microperimetric testing results were superimposed on the SW-AF image after image registration.

**Figure 1.**
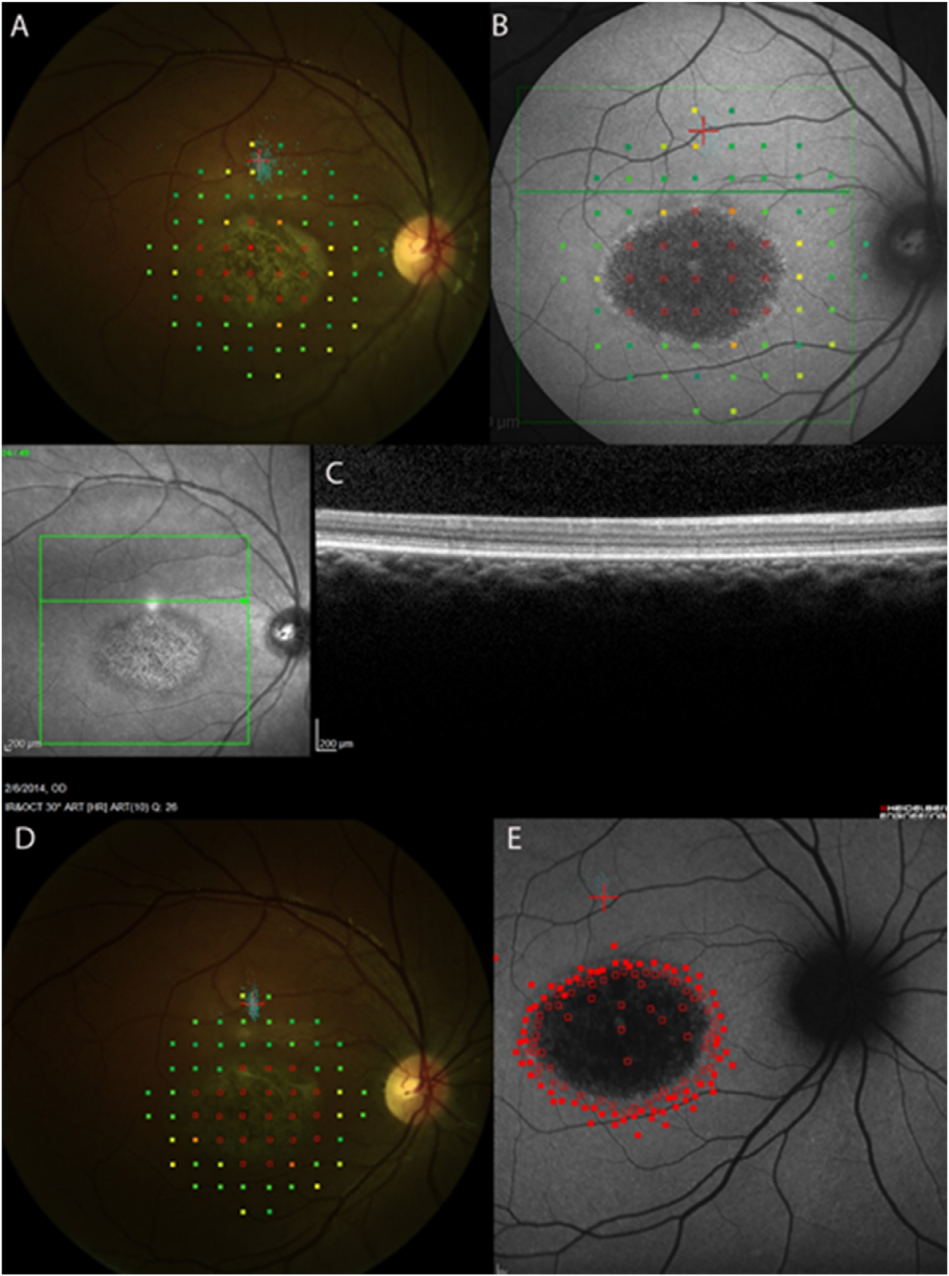
Microperimetry, SW-AF, and OCT findings at baseline and 24 months. Empty *red squares* indicate sites of dense scotoma. *Solid red squares* indicate that the brightest (0 dB) stimulus was seen. A pseudocolor map is used to indicate the retinal sensitivity, with *green squares* indicating that the dimmest stimuli (near 20 dB) were seen. The *small blue dots* show retinal sites viewing the fixation cross during testing. The 10-2 testing pattern consists of 68 points spaced at 2° intervals, and testing out to 10° eccentricity. (**A**) At baseline, fixation (*red cross and cluster of blue fixation points*) is approximately 4° (1200 µm) remote from the edge of the dense scotoma, measured by 10-2 threshold microperimetry. The distance was measured by using the 10-2 grid. The dense scotoma equals the dark lesion. (**B**) At baseline, the background autofluorescence pattern is uniform. (**C**) OCT at baseline shows intact EZ (FIL location shown by *green horizontal line*) in region between fixation and the edge of the scotoma. Thus the remote fixation is not explained by the OCT findings. (**D**) At 24 months, fixation remains remote from the edge of the dense scotoma. (**E**) At 24 months, dense scotoma mapping shows that the scotoma corresponds to the dark lesion, and the background autofluorescence pattern remains uniform. The brightest (0 dB) stimuli were used. *Solid squares* are where stimulus was seen. *Open squares* are where it was not.

**Figure 2.**
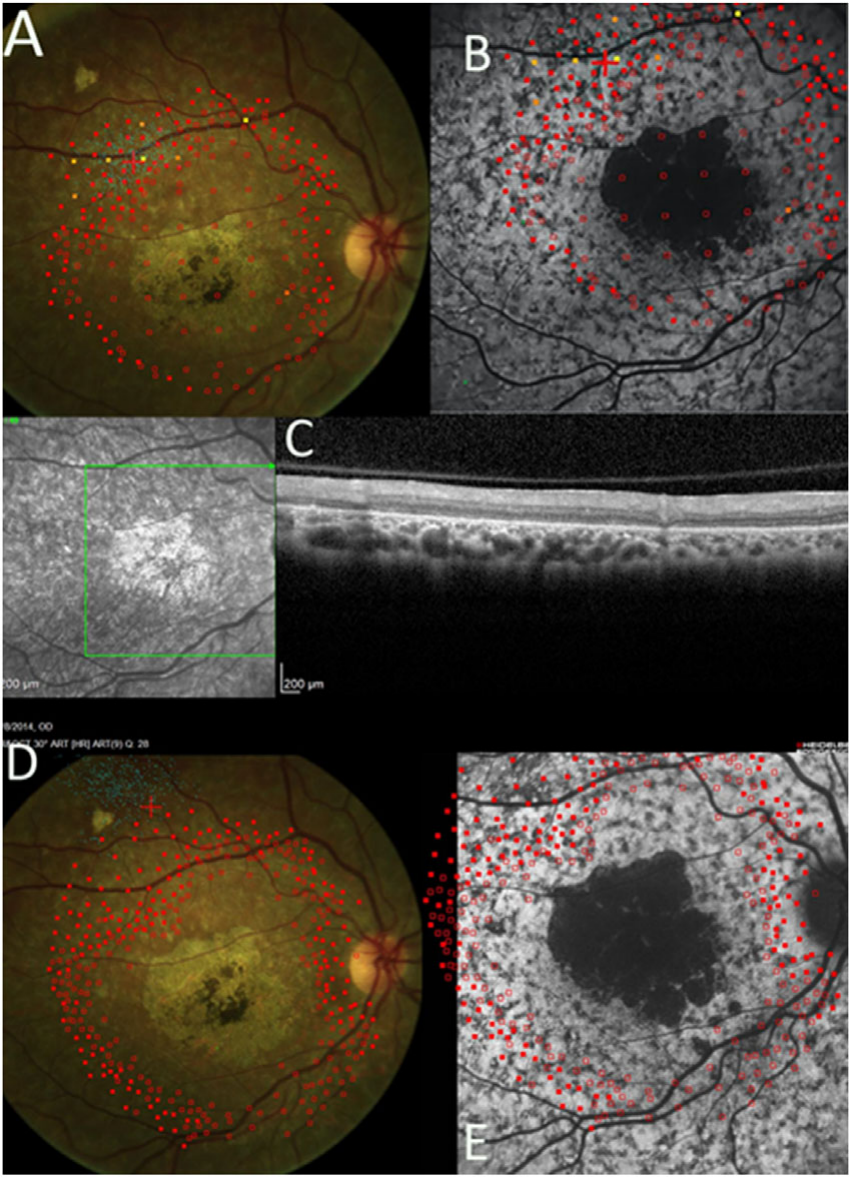
Dense scotoma mapping by microperimetry, SW-AF, and OCT findings at baseline and 24 months. *Empty red squares* indicate sites of dense scotoma. *Solid red squares* indicate that the brightest stimulus was seen. Other stimuli of lower intensity (*indicated by colors other than red*) were used in a few locations to measure the level of retinal sensitivity. The dense scotoma exceeded the standard 10-2 field, so that dense scotoma mapping was necessary to delineate the edge of the scotoma. (**A**) At baseline, fixation (*small blue dots*) was not stable, but was placed at the edge of the dense scotoma. (**B**) The microperimetry is overlaid on the SW-AF image. There is a mottled background pattern present. The dense scotoma greatly exceeds the dark lesion, and there are no particular features delineating the seeing from the nonseeing retinal areas. (**C**) The OCT scan was entirely within the nonseeing area and it was abnormal throughout. (**D**) At 24 months, the dense scotoma has enlarged. The fixation site is now remote from the edge of the dense scotoma. (**E**) At 24 months, the SW-AF image shows that the dark lesion has enlarged somewhat, but most of the mottled area remains unchanged in appearance.

**Figure 3.**
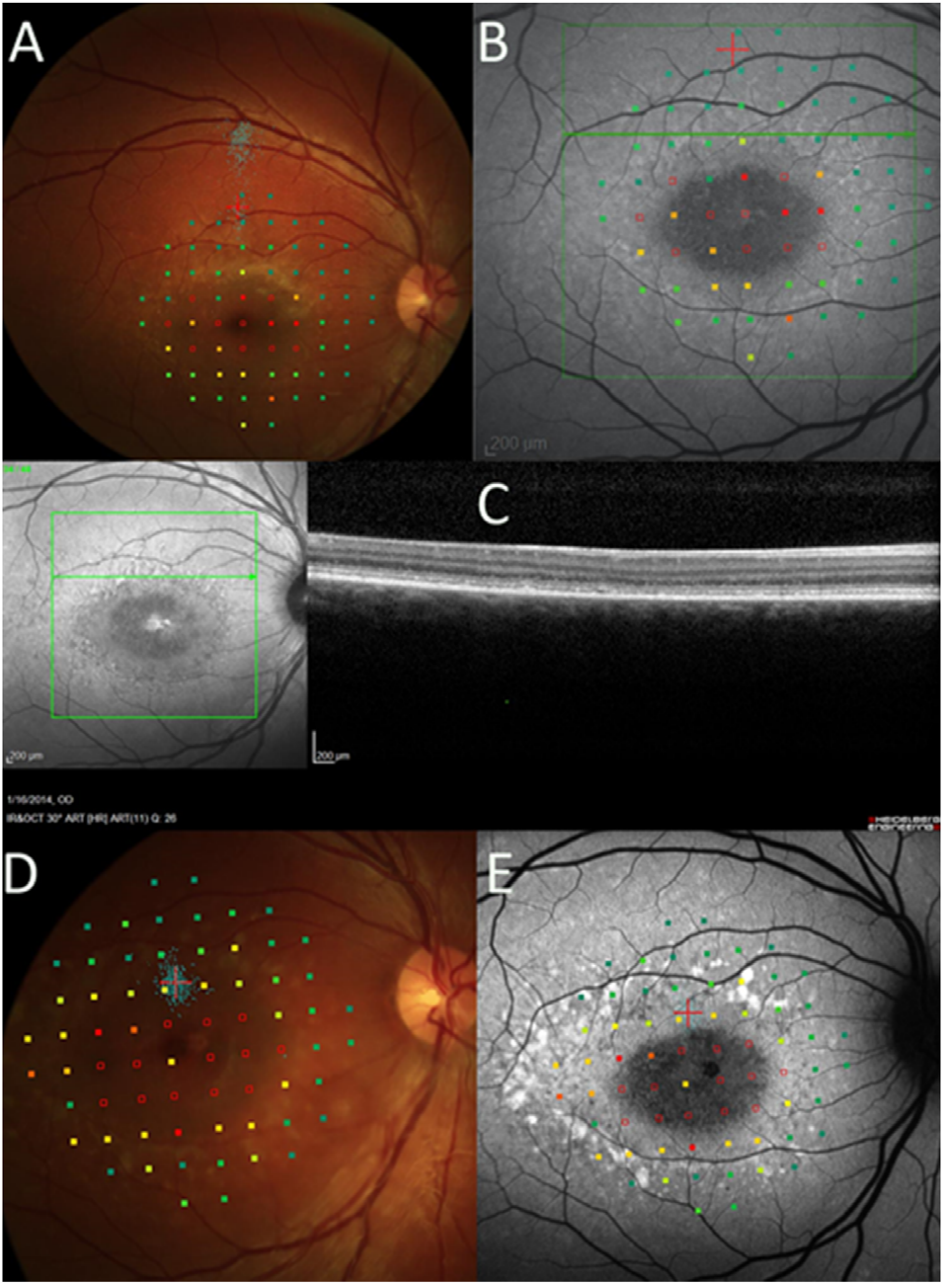
The 10-2 microperimetry, SW-AF, and OCT findings at baseline and 24 months. *Empty red squares* indicate sites of dense scotoma. *Solid red squares* indicate that the brightest (0 dB) stimulus was seen. A pseudocolor map is used to indicate the retinal sensitivity, with *green squares* indicating that the dimmest stimuli (near 20 dB) were seen. The *small blue dots* show fixation sites during testing. (**A**) At baseline, fixation was far removed from the edge of the dense scotoma (*blue dots*; more remote than where the fixation cross was originally positioned). (**B**) At baseline, fundus autofluorescence imaging, with microperimetry overlaid, shows that the dense scotoma does not exceed the dark central lesion. The background pattern is other pattern, with subtle hyperautofluorescent flecks but without hypoautofluorescent mottling. (**C**) The FIL of the OCT is near the edge of the dense scotoma. It does not explain why the fixation is remote. (**D**) At 24 months, fixation is placed at the edge of the dense scotoma. (**E**) At 24 months, fundus autofluorescence imaging shows that the hyperautofluorescent lesions are more prominent, but the pattern remains other pattern.

The resulting composite images, showing the SW-AF image with the microperimetry and FIL overlaid, were then classified for three features:

1) For eyes with eccentric fixation, the site of fixation was compared with the edge of the dense scotoma (i.e., the area that did not see the brightest stimuli). The distance between the fixation site and dense scotoma edge was measured from the closest edge of the one standard deviation bivariate contour ellipse area (68%) to the nearest dense scotoma edge. If fixation was more than 2° from the scotoma edge, fixation was classified as remote; otherwise it was classified as border (2° was used as the standard for the presence of a difference because the 2° metric was already on the image from the microperimetry 10-2 testing, and this was the testing interval). The classification is based on the dense scotoma edge, not on the edge of the dark area on autofluorescence imaging. For the eyes with remote fixation, the linear distance of fixation from the nearest scotoma edge was measured in degrees (1° equals 300 µm on the retina) using the MP-1 polar grid. The mean sensitivity in the intervening region between fixation and the dense scotoma was computed by averaging the sensitivity of the points to either side of the line connecting fixation to the nearest scotoma edge. The sensitivity of the fixation site was computed as the average of the four closest points surrounding fixation.
2) The extent of the dense scotoma was compared with the dark central lesion on SW-AF imaging. If the dense scotoma extended more than 2° past the lesion edge, the eye was classified as dense scotoma greater than lesion (greater scotoma); otherwise it was classified as equal to lesion (equal scotoma). The longest distance between the scotoma edge and the nearest edge of the lesion was measured in degrees using the MP-1 polar grid.
3) The retinal background outside the dark lesion on SW-AF imaging was defined into one of three categories. U, uniform (or uninvolved), meaning that the background looked essentially normal with no flecks or mottling (Fig. 1). M, mottled, meaning that there was mottled hypoautofluorescence throughout all or most of the field imaged (Fig. 2). O, other pattern, meaning that the background pattern showed primarily hyperautofluorescent flecks with little hypoautofluorescence, or that the background was uniform except for the region immediately surrounding the central lesion (Fig. 3).

The prevalence of these three characteristics and the relationship among them was assessed for the baseline visit. The findings at 24 months were compared with the baseline findings.

OCT scans were analyzed to determine if changes in photoreceptor-attributable reflectivity bands could account for the remote fixation or for the dense scotoma extending past the dark lesion. Starting from the lesion and proceeding superiorly, the first line with a fully intact EZ was identified. The location of this line (i.e., FIL) was compared with the fixation location and with the edge of the scotoma.

### Statistical Analysis

JMP software 15 (SAS Institute, Cary, NC, USA) was used. The Fisher's exact test and the χ^2^ test were used to compare characteristics. For most continuous data, an unpaired *t* test with equal variances was used. Microsoft Excel (Microsoft, Redmond, WA, USA) was used for the box and whisker plots.

## Results

Supplementary Table S1 provides the listing of patients included, together with age of onset, duration of disease, sex, visual acuity, classification by fixation, dense scotoma, background autofluorescence, genetic findings, and full-field electroretinographic classification for each patient. Table 1 provides a summary of the age at baseline, age at diagnosis, and duration of disease for the patients. Median age at the baseline visit was 26 years (range, 12–58 years). The median age of symptom onset or diagnosis was 16 years. Fifteen patients were diagnosed younger than 20 years. Median duration of disease was 8 years, with 3 having a duration greater than 15 years. There were 18 female and 7 male patients.

**Table 1.** Summary of Time Course of Disease

Age at Baseline Visit		Age at Diagnosis or Symptom Onset^*^		Duration of Disease	
Median 26, Range 12–58		Median 16, Range 6–41		Median 8 y, Range 1–25	
Age range (y)	# pts	Age range (y)	# pts	Range (y)	# pts
10–19	10	5–9	6	1–5	9
20–29	4	10–19	9	6–10	7
30–39	8	20–29	7	11–15	6
40–49	2	30–39	1	16–20	2
50–59	1	40–49	2	21–25	1

* The earlier date (diagnosis or symptom onset) was used, except when the diagnosis was made of genotypic Stargardt disease in an asymptomatic sibling of a diagnosed patient. In this case, the age of symptom onset was used for the sibling.

Each eye was classified by fixation pattern (border, remote, or central), whether the dense scotoma equaled or was greater than the dark lesion, and by the background autofluorescence pattern (uniform [U] Fig. 1, mottled [M] Fig. 2, or other pattern [O] Fig. 3). Table 2 provides a 2 × 4 table categorizing the eyes by their fixation (column classification) and dense scotoma properties (row classification). Within each cell of the table, the background autofluorescence pattern is provided for the eyes in that cell.

**Table 2.** Classification of Eyes by Fixation Pattern and Dense Scotoma (*n* = 49)

	Fixation at Edge of Dense Scotoma	Fixation Remote from Scotoma Edge	Fixation Central	Total
Dense scotoma = lesion	2 (4%)	23 (47%)	6 (12%)	31 (63%)
	2O	10M, 6U, 7O	2M, 4U	12M, 10U, 9O
Dense scotoma > lesion	5 (10%)	10 (22%)	3 (6%)	18 (37%)
	5M	10M	1M, 2O	16M, 2O
TOTAL	7 (14%)	33 (67%)	9 (18%)	49 (100%)
	5M, 2O	20M, 6U, 7O	3M, 4U, 2O	28M, 10U, 11O

Each entry is the number of eyes, the percentage of all eyes, and the background pattern of autofluorescence.

M, mottled hypoautofluorescence pattern of background (*n* = 28, 57% of all eyes);

U, uninvolved or uniform pattern of background (*n* = 10, 20% of all eyes);

O, other pattern, hyperautofluorescent flecks without mottled hypoautofluorescence, or uninvolved pattern except in junctional zone (*n* = 11, 22% of all eyes).

Fixation was remote from the scotoma edge in 33 eyes (67%) (Figs. 1, 3A, 3B), was at the border of the scotoma in 7 eyes (14%) (Figs. 2A, 2B), and was central in 9 eyes (18%). Thus of the 40 eyes with eccentric fixation, 33 (82%) had fixation remote from the edge of the dense scotoma. In addition, five of the seven eyes with fixation at the border of the scotoma had the scotoma significantly larger than the dark lesion (Fig. 2). Thus only two eyes of one patient exhibited fixation at the edge of the dark lesion.

Table 3A presents the distance of fixation from the dense scotoma in the 33 eyes with remote fixation. Twenty-four of the eyes (73%) had fixation at least 5° (1500 µm) from the dense scotoma edge. Table 3B provides the mean sensitivity in the intervening retina between the dense scotoma and the fixation site. The MP-1 measures a range of sensitivity from 0 to 20 dB. For six eyes, the dense scotoma exceeded the 10-2 field in which threshold testing was performed. Of the remaining 27 eyes, 8 (30%) had sensitivities of 13 dB or better. There was no significant difference in the mean sensitivity of the intervening retina between eyes fixating within 5° of the dense scotoma edge and eyes fixating more than 5° from it (mean sensitivity 11.4 for the within 5° group, 11.5 for the >5° group, t = 0.04, *P* = 0.97). The ProgStar study defined sensitivities of 12 dB or better to be not significantly abnormal.^17^ Thus there generally was not markedly reduced sensitivity in the intervening retina to explain the cause of the remote fixation. In addition, for most eyes the remote fixation site had a sensitivity comparable to the intervening region. For the 15 eyes in which fixation was within the 10-2 grid so that sensitivity could be assessed, 9 had sensitivity at fixation that was at most 2 dB better than the intervening region. Four eyes had sensitivity at fixation that was between 2 and 4 dB better. Only two eyes had sensitivity at fixation significantly better (6 and 8 dB better).

**Table 3.** Quantification of Remote Fixation

Fixation Remote from Scotoma Edge (*n* = 33)	
A. Distance of fixation from scotoma edge	# eyes
3°–4°	9
5°–6°	14
≥7°	10
B. Mean sensitivity in intervening retina (dB)	# eyes
6-9	9
10-12	10
13-15	4
16-19	2
20	2
Could not measure^*^	6

* When fixation was outside the area tested with the 10-2 grid, the sensitivity values were not available in the unmeasured area.

The dense scotoma exceeded the dark SW-AF lesion (called the lesion, or the dark lesion) in 18 eyes (37%) (Table 2, Fig. 2), including 5 of the 7 eyes with border fixation, 10 of 33 eyes with remote fixation, and 3 of 9 eyes with central fixation. Thus it was present in all fixation patterns (χ^2^ 4.12, *P* = 0.13). Table 4 presents the quantification of the linear extent of the dense scotoma exceeding the lesion. There was a median excess scotoma of 5° to 6°, with 6 eyes having an excess scotoma length of 7° or more.

**Table 4.** Length of Dense Scotoma Outside Dark Lesion

Dense Scotoma Exceeding Lesion (*n* = 19)	
Length of excess dense scotoma	# eyes
3°–4°	3
5°–6°	8
≥7°	6
Could not measure	2

**Figure 4.**
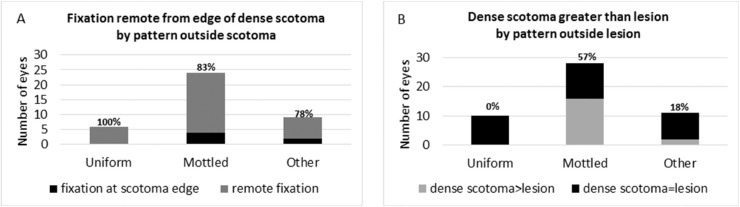
(**A**) The association of fixation remote from the edge of the scotoma with the background pattern of autofluorescence. One hundred percent of eyes with the uniform pattern, 83% of eyes with the mottled pattern, and 78% of the eyes with the other pattern had remote fixation. There is no significant difference between the mottled pattern and the uniform plus other patterns by the Fisher's exact test. (**B**) The association of dense scotoma exceeding the dark lesion with the background pattern of autofluorescence. None of the eyes with the uniform pattern, 57% of eyes with the mottled pattern, and 18% of eyes with the other pattern had dense scotoma exceeding the dark lesion. The difference between the mottled pattern and the uniform plus other patterns is significant by the Fisher's exact test.

**Figure 5.**
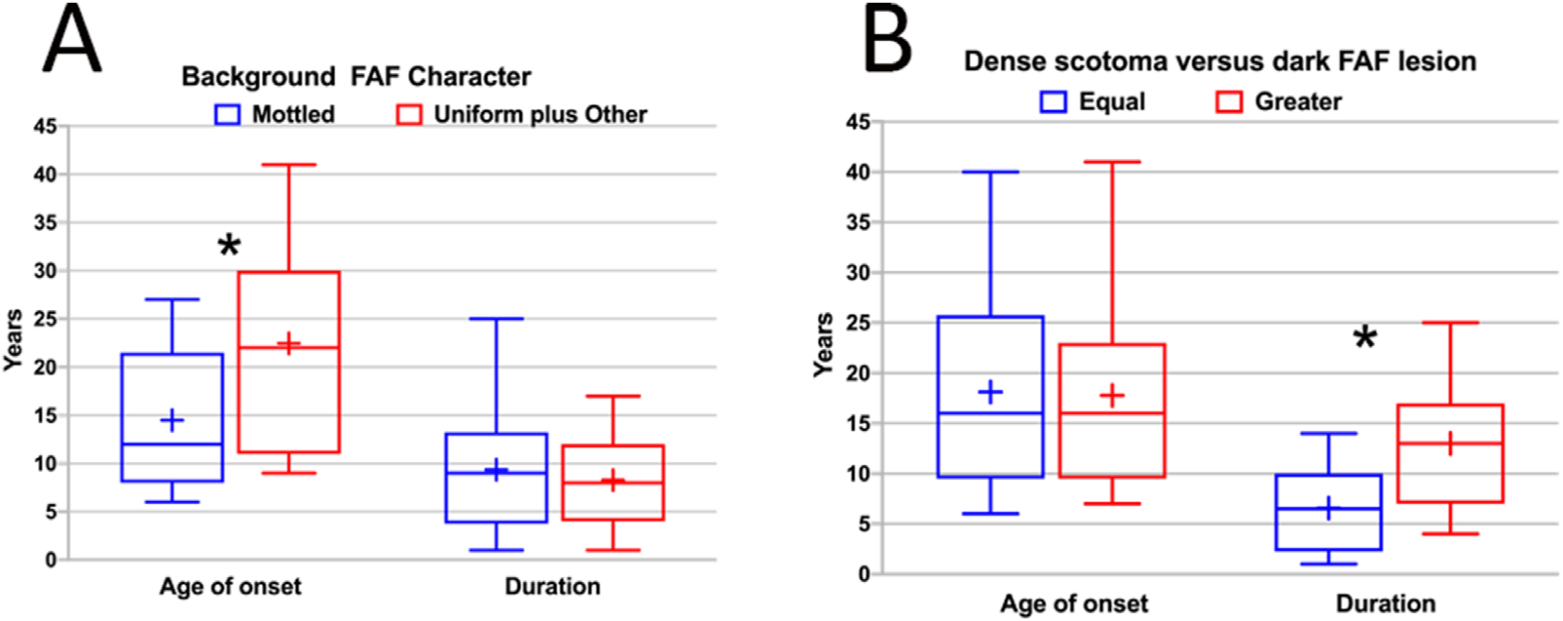
(**A**) The relationship of the background autofluorescence character (mottled vs. uniform plus other patterns) as a function of approximate age of onset of disease and as a function of duration of disease. The *blue box* is the mottled group, and the *red box* is the uniform plus other group. The horizontal line is the median, the “+” is the mean, and the height of the box represents the 25th to 75th percentiles. The mottled group has a significantly earlier age of onset of disease than the other plus uniform group. The duration of disease distribution was essentially identical in the two groups. The *asterisk* signifies the statistically significant difference. (**B**) Dense scotoma characteristic (equal in size to dark lesion vs. greater than dark lesion) as a function of approximate age of onset of disease and as a function of duration of disease. The *blue box* is the equal scotoma group, and the *red box* is the greater scotoma group. The horizontal line is the median, the “+” is the mean, and the height of the box represents the 25th to 75th percentiles. The age of onset distribution is essentially identical in the two groups. The duration of disease was significantly longer in the greater dense scotoma group. The *asterisk* signifies the statistically significant difference.

The background pattern and its relationship to these findings were assessed (Table 2, Figs. 4 and 5). The mottled background pattern was the most common; it was present in 28 eyes (57%). The uniform pattern was present in 10 eyes (20%), and the other pattern was present in 11 eyes (22%). The mottled pattern was more strongly associated with having a dense scotoma exceeding the lesion than were the uniform or other patterns (16/28 (57%) of mottled, 2/11 (18%) of other, and 0% of uniform (Fisher's exact test, *p* = 0.0008 for mottled versus [other plus uniform]) (Fig. 4B). However, there was no significant difference between the three background categories in terms of remote fixation (χ^2^ 2.32, *P* = 0.31) (Fig. 4A).

Age of onset was related to the background pattern, with mean age of onset 14.5 years for mottled, and 22.5 years for the other and uniform patterns combined (t = –2.15, *P* = 0.04; Fig. 5A), whereas the duration of disease had no significant association with background pattern (t = −0.44, *P* = 0.67). For the remoteness of fixation, the age of onset was also significant, with mean age of onset 14.5 years for remote, 24.3 years for border, and 26 years for central (*F* = 4.2, *P* = 0.03). Duration was not different among the groups.

In contrast, the dense scotoma pattern has a different dependence (Fig. 5B). There was no difference in age of onset between the equal scotoma group (mean age 18.1 years) and the greater scotoma group (17.8 years) (t = –0.08, *P* = 0.93). However, the dense scotoma pattern is strongly associated with duration, with mean duration of disease 6.6 years for the equal scotoma group, and 13 years for the greater scotoma group (t = –3.00, *P* = 0.0065).

The eyes with central fixation were essentially identical to the eyes with eccentric fixation in terms of the equal scotoma or greater scotoma classification (67% of eyes with central fixation had equal vs. 62% of eyes with eccentric fixation; χ^2^ = 0.06, *P* = 0.81.) There was a trend toward a difference in background pattern, with 33% of eyes with central fixation having mottled versus 63% of eyes with eccentric fixation, but this was not statistically significant (χ^2^ = 2.63, *P* = 0.10.)

The visual acuity of the better-seeing eyes of those patients without central fixation had a mean and median of 20/160 (0.90 logMAR), and a range of 20/80 to 20/320 (0.60 to 1.20 logMAR). Within this limited visual acuity range, there was no significant difference in visual acuity between eyes with border versus remote fixation (0.85 logMAR for border, 0.90 for remote; t = 0.58, *P* = 0.57), between eyes with dense scotoma equaling versus exceeding the dark lesion (0.87 logMAR for equal, 0.94 for greater; t = 1.13, *P* = 0.27), or between eyes with mottled versus nonmottled (other plus uniform) background pattern (0.92 logMAR for mottled, 0.85 for other plus uniform, t = –1.16; *P* = 0.26.)

The two eyes of each patient were concordant in fixation, dense scotoma, and background autofluorescence classification at baseline, except for one patient who had central fixation in one eye, and had border fixation in the fellow eye. The classification of most eyes did not change over the 24-month follow-up interval. At the 24-month follow-up, all eyes but one eye of each of two patients had the same classification as at the baseline visit. One eye fixated remotely at baseline but at the scotoma edge at 24 months. (Fig. 3). The other eye fixated at the edge of the dense scotoma at baseline, with a dense scotoma far exceeding the dark lesion, but fixated remote from the edge of the scotoma at 24 months (Fig. 2).

Supplementary Table S1 provides the mutations found for each patient. Ten patients had two missense mutations, and 11 had one missense and one splice site mutation. Patients in these two groups had essentially the same distribution of remote versus border fixation (Fisher's exact test *P* = 0.47) and equal versus greater dense scotoma (Fisher's exact test *P* = 0.66). However, for the background patterns there was a difference that was not statistically significant (5/10 patients with two missense mutations had mottled, whereas 9/11 patients with one missense and one splice site mutation had mottled, Fisher's exact test *P* = 0.18). Two patients with one frameshift and one missense mutation had the equal dense scotoma category, and did not have the mottled pattern. The remaining two had deep intronic mutations with uncertain classification.

Supplementary Table S1 provides the electroretinographic categorization for 23 of the patients. Fifteen had normal ERGs, and were group 1 by the Lois definition,^19^ 5 had both scotopic and photopic responses reduced (group 3 by the Lois definition), and 3 had scotopic responses reduced but photopic responses normal. There was no obvious difference and no significant difference between group 1 and group 3 in terms of remoteness of fixation (Fisher's exact test *P* = 0.46) or relationship of dense scotoma to the dark lesion (Fisher's exact test *P* = 0.79). For the background, 7 of the 15 group 1 eyes (47%) had mottled, versus 4 out of 5 group 3 patients (80%), but this was not statistically significant (Fisher's exact test *P* = 0.22).

For the OCT analysis, for the 7 eyes with border fixation, 2 had the FIL within 2° of fixation, 3 had nasal or temporal fixation, so the correlation could not be performed, and 2 could not be analyzed because the scotoma and fixation extended well past the OCT field tested. For the 33 eyes with remote fixation, 18 had the FIL within 2° of fixation, thus providing an explanation for the remote fixation despite good sensitivity. Six eyes had the FIL 2° to 4° before fixation, that is, closer to the edge of the scotoma, so that the further remoteness of fixation was not fully explained. The remaining 12 eyes did not provide information, with 4 having nasal or temporal fixation, 1 with no fixation map, and 7 with no information because the scotoma extended well past the OCT field tested. Thus of the 24 eyes with remote fixation that could be analyzed, 18 (75%) had the FIL near the fixation site, and 6 (25%) fixated further peripheral than the OCT would explain.

The relationship of the FIL to the edge of the dense scotoma was studied. For the equal scotoma group (in which the scotoma corresponded to the dark lesion), two eyes had OCT fields that did not extend into seeing retina, and were thus not informative, and one could not be measured. Of the remaining 28 eyes, 16 eyes (57%) had the FIL within 2° of the scotoma edge, 10 (36%) had the FIL 2° to 4° into the seeing retina, and 2 (7%) had the FIL 4° to 8° into the seeing retina. Thus 43% of eyes had OCT abnormality extending into seeing retina, so that the OCT abnormality did not define the edge of the dense scotoma.

For the greater scotoma group (in which the scotoma exceeded the dark lesion), 12 eyes had OCTs that were not informative (because the OCT field did not extend into seeing retina), and one had the FIL 5° within the dense scotoma. For the remaining 5 eyes, 2 had the FIL 2° to 4° into seeing retina, and 3 had the FIL 4° to 8° into seeing retina. Thus in the greater group the OCT may not help to define the edge of the dense scotoma, either because the scotoma extended so far peripherally, or because the OCT abnormality extended into the seeing retina.

## Discussion

This study, to our knowledge, is the first study to quantify the total extent of dense scotoma using dense scotoma mapping, and to correlate these findings with SW-AF and OCT observations in STGD1. Our study shows evidence of photoreceptor dysfunction, reflected by the sites not being used for fixation and/or by the presence of a dense scotoma. In 82% of eyes, fixation was placed remote from the edge of the dense scotoma, unexplained given the good sensitivity in the intervening retina. In 37% of eyes, the dense scotoma extended past the edge of the dark, hypoautofluorescent, lesion. Our study presents evidence of EZ disruption extending far past the edge of the dark SW-AF lesion and far past the edge of the dense scotoma, even when the dense scotoma exceeds the dark lesion. The EZ abnormalities account for the remote fixation found in STGD1 despite intervening seeing retina in 75% of cases. In addition, eyes with dense scotomas extending past the edge of the dark SW-AF lesion^12^^,^^14^ have EZ band abnormalities not only in the area of dense scotoma, but extending into seeing retina. The mottled background pattern was most likely to have the dense scotoma exceeding the dark lesion. Gomes et al.^14^ showed similar extension of OCT abnormalities outside the dark lesion, but these were generally small and limited to the perilesional area. Verdina et al.^15^ reported on the presence of remote fixation, averaging 5°, in 67% of eyes, with OCT abnormality in the intervening retina of 77% of these patients.

Thus there is evidence of photoreceptor dysfunction at eccentricities that neither the dense scotoma nor OCT (nor SW-AF) capture fully. Structural changes visible in en face images derived from wide-field swept-source OCT have revealed correspondence between the abnormal reflective region in the IS/OS slab and the abnormal SW-AF area that included both hypoautofluorescent and hyperautofluorescent zones,^20^ although the areas involved were not as large as in this study (Fig. 2). Adaptive optics reflectance and SW-AF imaging suggests that there may be photoreceptor abnormality in areas of only subtle RPE changes.^21^

NIR-AF that visualizes RPE atrophy using a loss of the melanin signal yields a larger dark lesion than does SW-AF, and in some cases corresponded to or even exceeded the zone of EZ loss in OCT scans.^22^^,^^23^ Near infrared SW-AF may be a better measure of RPE integrity, and may show larger areas of involvement, but whether it captures the full dense scotoma remains to be determined. Cideciyan et al.^24^ used the leading disease front, defined as the peripheral edge of the heterogeneous or dark homogeneous lesion on NIR-AF, to measure progression of disease, but it does not directly correspond to visual function.

In our study, although 82% of all eyes with eccentric fixation had remote fixation, there was still a dependence of remote fixation on the age of onset of the disease. One cannot rule out the possibility that there is some cortical factor involved in setting the fixation at a remote site. We are not aware of information relating to remoteness of fixation in other young onset macular dystrophies.

There are several limitations to our study. The FIL may not correspond to the exact location of the beginning of the fully intact EZ, so that the fully intact EZ may actually occur closer to the dense scotoma or fixation point than is captured by the OCT analysis performed. A more thorough analysis of the EZ map, extending more peripherally, would be beneficial. We had a higher percentage of eyes with the mottled, also called “heterogeneous,” pattern (57%) than in the ProgStar study as a whole (32%),^25^ although other studies had an even higher rate (90% in the Fujinami et al.^26^ study; the uniform and other categories were combined in the Fujinami et al. study as homogeneous, whereas they were assessed separately for many measures in this article). It may be the case that for eyes without the mottled pattern, the dark lesion correlates closely enough with the scotoma to be useful in quantifying the dense scotoma. Also, our study included only the ProgStar subjects at our center. Although the other centers did not use dense scotoma mapping, for many eyes at our center the fixation site, even though remote, was still within the 10-2 field, so that for these patients the remoteness of fixation and the sensitivity of the intervening retina could be assessed. In terms of assessing fixation, it would be more informative to allow the patient to fixate freely, keeping the cross in the center of the field, so that the sensitivity at fixation and fixation stability could be better measured. However, fixating at the center of the field would in many cases have moved the fovea out of the testing field and would have limited the microperimetry data collection, which was the most important aspect of the testing. No patient showed evidence of having two eccentric loci for fixation, although this is also a theoretical problem in terms of testing fixation.

## Conclusions

A better understanding of photoreceptor dysfunction outside the dark lesion is important in several respects. An intriguing question is whether disease may be reversible in these ambiguous retinal areas that do not yet show RPE atrophy or photoreceptor loss yet have significant functional impairment. STGD1 is different from geographic atrophy and other atrophic macular disorders, in which the fundus photograph, infrared image, and fundus autofluorescence all define regions of atrophy that correspond to the dense scotoma,^27^ and there are no ambiguous areas. A reduction of real-time bisretinoid formation in the photoreceptors may be amenable to treatments that decrease further bisretinoid accumulation, even if the already lipofuscin-laden RPE is not helped by such intervention. Future treatments might be able to look at the impact on these regions as a means of improving visual function in these patients. One would like to be able to use a combination of imaging characteristics and functional measures to follow progression of the disease because either one alone is insufficient, as shown by this work.

## Supplementary Material

Supplement 1
